# Association of c.56C > G (rs3135506) Apolipoprotein A5 Gene Polymorphism with Coronary Artery Disease in Moroccan Subjects: A Case-Control Study and an Updated Meta-Analysis

**DOI:** 10.1155/2020/5981971

**Published:** 2020-08-04

**Authors:** Imane Morjane, Hicham Charoute, Sanaa Ouatou, Lamiae Elkhattabi, Houda Benrahma, Rachid Saile, Hassan Rouba, Abdelhamid Barakat

**Affiliations:** ^1^Laboratory of Genomics and Human Genetics, Institut Pasteur Du Maroc, Casablanca, Morocco; ^2^Laboratory of Biology and Health, Faculty of Sciences Ben M'Sik, Hassan II University of Casablanca, Casablanca, Morocco; ^3^National Reference Laboratory (LNR), Faculty of Medicine, Mohammed VI University of Health Sciences (UM6SS), Casablanca, Morocco

## Abstract

**Purpose:**

Coronary artery diseases (CAD) are clinical cardiovascular events associated with dyslipidemia in common. The interaction between environmental and genetic factors can be responsible for CAD. The present paper aimed to examine the association between c.56C > G (rs3135506) APOA5 gene polymorphism and CAD in Moroccan individuals and to perform an association update meta-analysis.

**Materials and Methods:**

The c.56C > G variant was genotyped in 122 patients with CAD and 134 unrelated controls. Genetic association analysis and comparison of biochemical parameters were performed using *R* statistical language. In addition, a comprehensive meta-analysis including eleven published studies in addition to our case-control study results was conducted using Review Manager 5.3. Publication bias was examined by Egger's test and funnel plot.

**Results:**

The case-control study data showed that the c.56C > G polymorphism was associated with CAD susceptibility under codominant (*P*-value = 0.001), recessive (*P*-value <0.001) and log-additive (*P*-value = 0.008) inheritance models. In addition, this polymorphism was significantly associated with increased levels of systolic and diastolic blood pressures, triglycerides, glycemia, and total cholesterol. Furthermore, meta-analysis showed a significant association between the c.56C > G gene polymorphism and increased risk of CAD under recessive (OR = 3.39[1.77–6.50], *P* value <0.001) and homozygote codominant (OR = 3.96[2.44–6.45], *P* value <0.001) models.

**Conclusion:**

Our case-control study revealed a significant association between c.56C > G polymorphism and CAD in the Moroccan population. In addition, meta-analysis data supported the implication of this polymorphism in CAD susceptibility.

## 1. Introduction

Coronary artery disease (CAD) is the most common cardiovascular disease. It is caused by ischemia and hypoxia [1] due to the formation of plaque which hardens and narrows the coronary arteries, and it is the major cause of death worldwide, accounting for nearly 7 million deaths annually [2]. CAD is considered as the most common cause of death among both men and women over the age of 50 [3]. Environmental factors associated with CAD include obesity, alcohol intake, smoking, diabetes, hypertension, and dyslipidemia [4]. In addition, genetic factors contribute to the occurrence and development of CAD. Elevated triglyceride (TG) and low HDL-C levels are characteristic of dyslipidemia, which is associated with high risk of cardiovascular events [4, 5]. The apolipoprotein A5 (apoAV) encoded by the APOA5 gene [6, 7] that belongs to the APOA1/C3/A4/A5 gene cluster on chromosome 11q23 regulates lipoprotein lipase (LPL) activity [8, 9]. New physiological roles of apoAV have been recently elucidated, such as control of chylomicron production in the intestine and TG accumulation in adipose tissue [10]. Epidemiological studies showed a strong correlation between increased TG concentrations and the elevation of coronary artery disease risk and more importantly with all-cause mortality [3, 11–14]. Moreover, the deficiency of the endogenous APOA5 gene leads to 4-fold increased serum TG concentrations in mice and were decreased by 65% by overexpression of the human APOA5 gene [6, 15]. The role of APOA5 gene polymorphisms in CAD has been investigated in many studies and meta-analyses, the results are controversial [10, 16–19]. This discrepancy could be explained by the differences between ethnic populations that can contribute to the variation of prevalence rates of CAD. Furthermore, new studies are continuously being performed to identify whether APOA5 polymorphisms are associated with cardiovascular diseases occurrence [20–23]. In addition, accumulated data make it possible to conduct cumulative meta-analysis and assess the effects of APOA5 polymorphisms. To date, more than 13 polymorphic sites of APOA5 have been reported [24–26]. Indeed, several studies in different populations have found the association between the APOA5 polymorphisms and the risk of CAD [18, 20, 27]. The APOA5 gene polymorphism, c.56C > G variant located in the exon3, is of a special interest and has been reported to be associated with an increased risk of CAD in multiple ethnic populations probably through its association with hypertriglyceridemia [3, 12]. However, there are also discrepant reports of no association between this polymorphism and CAD risk [28, 29].

In order to evaluate the associations between APOA5 gene c.56C > G polymorphism and CAD, we performed a case-control study in a Moroccan population. In addition, we conducted a meta-analysis to confirm the contribution of this APOA5 polymorphism to CAD in various ethnic populations.

## 2. Material and Methods

### 2.1. Study Population

Study participants were recruited in the Medical Biology Center of Pasteur Institute of Morocco and Ibn Rochd Hospital. The present study involved 122 patients (73 men and 49 women, mean age 60.70 ± 12.86) with coronary artery disease confirmed by coronary angiography, defined as the presence of at least one coronary artery with ≥50% organic stenosis. The study recruited also 134 unrelated non-CAD controls (42 men and 92 women, mean age 50.78 ± 12.08). All subjects were Moroccan adult volunteers and all provided their written informed consents. Participants completed a health and lifestyle questionnaire including sociodemographic characteristics, medical history, physical activity, medication intake, and alcohol and tobacco use. Clinical, biochemical, anthropometric parameters, and CAD risk factors (hypertension and diabetes mellitus) were assessed in both patients and controls. Hypertension was defined as systolic blood pressure >140 mmHg or/and diastolic blood pressure >90 mmHg or using antihypertensive drugs. Type 2 diabetes mellitus was diagnosed according to World Health Organization criteria. All women were nonpregnant and nonlactating. Healthy subjects had normal biochemical variables, were nonsmoking, and without history of CAD.

### 2.2. Biochemical Measurements

Blood samples were collected in EDTA tubes after at least 12 hours of overnight fast, centrifuged, and stored until subsequent laboratory analysis. Fasting plasma glucose, total cholesterol (TC), triglycerides (TG), and high-density lipoprotein cholesterol (HDL-C) levels were determined using the VITROS automate analyzer (5.1 FS Chemistry System). Low-density lipoprotein cholesterol (LDL-C) level was calculated according to the Friedewald formula.

Genomic DNA was isolated from peripheral leukocytes using the standard proteinase *K* digestion followed by phenol-chloroform extraction and ethanol precipitation. The c.56C > G polymorphism was genotyped by PCR-RFLP analysis. PCR reactions were performed in a Biometra thermal cycler, using Taq polymerase (Bioline), and restriction fragments were analyzed by agarose gel migration (3%). Primers, PCR conditions, and RFLP analysis were performed according to a previously published protocol [30].

PCR of 157 bp fragment of the 56C > G polymorphism was performed using the following oligonucleotides: Forward: 5′- GGC TCT TCT TTC AGG TGG GTCTCCG -3′ and reverse: 5′- GCC TTT CCG TGC CTG GGT GGT -3′. The PCR started with an initial denaturing at 96°C for 5 min, followed by 30 cycles of 96°C for 30 s, 64°C for 30 s, and 72°C for 45 s and then a final extension of 72°C for 10 min. The PCR products were digested for 2 hours at 65°C with TaqI restriction enzyme: the 56C allele presents a TaqI restriction site which is suppressed in the 56G allele [31]. All molecular analyses were performed in the Genomics and Human Genetic Laboratory at Pasteur Institute of Morocco.

### 2.3. Literature Search Strategy for the Meta-Analysis

We conducted a comprehensive literature search using PubMed. Articles published up to January 2020 were searched using the following key words: (“coronary heart disease” OR “coronary artery disease” OR “myocardial infarction”) AND (“APOA5” OR “apolipoprotein A5” OR “C56G” OR “S19W” OR “rs3135506”) AND (“polymorphism” OR “SNP” OR “variant”). References cited in the identified articles were also analyzed to avoid missing any potential articles from the initial search. Data from studies were included in our meta-analysis only if the study met the following criteria: (1) only CAD as the outcome and the investigated variant should be c.56C > G exclusively or in the same study with other polymorphisms; (2) only the case-control studies were considered; (3) the genotype distribution of CAD cases and controls can be obtained from articles directly or by calculation; (4) the methods of data collection and analysis should be statistically acceptable; and (5) publications that presented data allowing such outcome to be derived were also included.

### 2.4. Statistical Analysis

Clinical and biochemical parameters normally distributed were expressed as means ± standard deviation (SD), and nonnormally distributed data were expressed as median (Interquartile range) while qualitative parameters were expressed as frequencies. Student's *t*-test was applied for comparison of quantitative traits that follow a normal distribution. Otherwise, we used the Mann–Whitney test. The Hardy–Weinberg equilibrium (HWE) analysis of the genotyped SNP was performed using an exact test, available in the R package “compareGroups.” The R package “SNPassoc” was used to examine the association between CAD and APOA5 genotypes. A *P* value less than 0.05 was considered statistically significant.

Furthermore, Review Manager 5.3 (The Cochrane Collaboration) was used to elaborate meta-analysis results. Heterogeneity between studies was assessed using the chi-square-based *Q*-test, and the significance was fixed to *P* < 0.10. The inconsistency index *I* [2] was also calculated to evaluate the variation caused by the heterogeneity. The possible publication bias was estimated using funnel plot. The R software was used to calculate *P* values of Egger's test.

## 3. Results

### 3.1. Subjects' Characteristics

Clinical characteristics and lipid parameters of patients with CAD and controls are presented in Table 1. As expected, triglycerides level (TG), glycemia (Gly), systolic blood pressure (SBP), and LDL-C were significantly higher among CAD patients than controls (*P* values <0.001). There were also significant differences in total cholesterol (TC) and diastolic blood pressure (DBP). No significant differences were found in body mass index (BMI) and HDL-C between both groups.

### 3.2. Association Analysis

We performed a case-control-based association analysis of the c.56C > G polymorphism with CAD (Table 2). The genotype distribution of the studied variant was in HWE (*P* value >0.05). Logistic regression analysis adjusted for sex, age, BMI, and smoking showed a significant association between the c.56C > G polymorphism and CAD susceptibility under co-dominant, recessive, and log-additive models.

### 3.3. Comparisons of Clinical and Biochemical Parameters between APOA5 Genotypes

We compared biological and clinical traits between APOA5 genotypes under homozygote and heterozygote codominant, dominant, and recessive genetic models. The APOA5 c.56C > G polymorphism was significantly associated with increased levels of systolic and diastolic blood pressure, triglycerides, glycemia, and total cholesterol (Table 3).

### 3.4. Meta-Analysis

**Figure 1:**
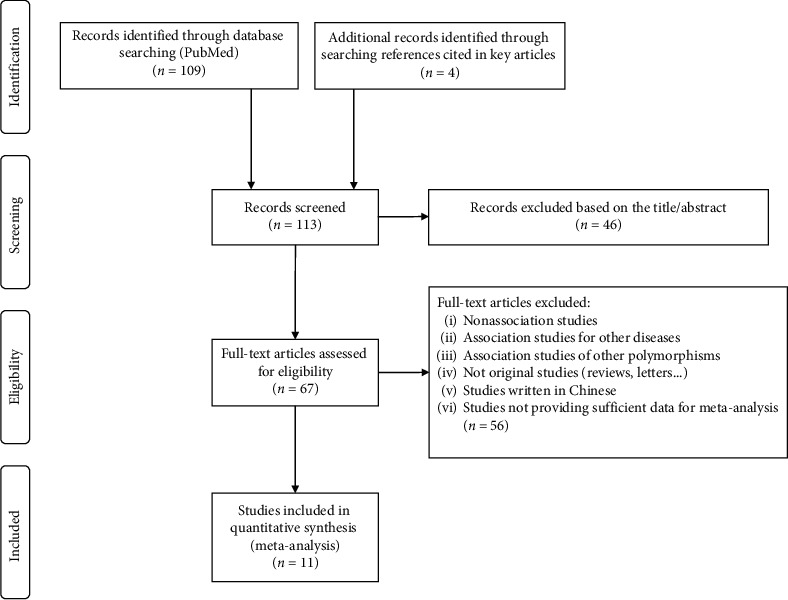
Flow chart of the study selection process for eligible studies.

The literature search identified 109 potentially relevant articles, the publication date of these studies ranged from 2004 to 2018, forty-six of which were excluded after initial screening of title and abstract. The full text of remaining 67 studies was reviewed, and 56 additional articles were excluded because they did not meet the inclusion criteria. Finally, 11 studies were included in our meta-analysis in addition to our case-control study, with a total of 5705 CAD patients and 7242 controls. A flow chart of study selection is shown in Figure 1 and characteristics of included are presented in Table 4.

Meta-analysis of the 12 genetic association studies and publication bias analyses are summarized in Table 5. The c.56C > G polymorphism was significantly associated with genetic predisposition to CAD under recessive mode of inheritance (OR = 3.39; 95% CI = [1.77–6.50]; *P* value <0.001). Moreover, using the homozygote codominant model, the GG genotype was significantly associated with susceptibility to CAD compared to the CC genotype (OR = 3.96; 95% CI = [2.44–6.45]; *P* value <0.001). A random-effect model was used to estimate pooled odds ratios if considerable heterogeneity was detected (*Q*-statistic: *P* < 0.10 or *I*^2^ > 50%). Otherwise, a fixed-effect model was used (*Q*-statistic: *P* > 0.10 and *I*^2^ < 50%). No significant publication bias was shown using both funnel plot analyses and Egger's test in all tested genetic models.

## 4. Discussion

Coronary artery disease (CAD) is a multifactorial disease in which atherosclerotic plaque accumulates inside the coronary arteries resulting in myocardial infarction and mortality. Hypertriglyceridemia is significantly associated with the increased risk of CAD, as elevated levels of triglycerides (TG) may lead to plaque formation [39–41]. The APOA5/4/C3/A1 gene cluster on chromosome 11q23 plays an important role in TG regulation; particularly variants occurring in the APOA5 gene have been extensively analyzed for their relationship with TG metabolism and cardiovascular diseases susceptibility [34, 36, 42]. Therefore, the present study was designed to examine the implication of the c.56C > G APOA5 variant as a coronary artery disease risk factor in the Moroccan population.

Logistic regression analysis adjusted for possibly confounding factors (sex, age, BMI, and smoking) showed a significant association between c.56C > G APOA5 variant and coronary artery disease. Various studies were performed in different populations in order to assess the association between this variant and CAD, but the results are conflicting. Consistent with our findings, the c.56C > G SNP was significantly associated with increased risk of CAD in a previous study on Chinese patients [33]. Similarly, a significant susceptibility to CAD was detected in subjects from Czech Republic carrying the rare allele of the c.56C > G SNP [32]. In contrast, two studies including patients of Italian and French origins failed to detect a relationship between c.56C > G SNP risk allele and CAD [28, 35].

Currently, the biological mechanisms by which the c.56C > G (S19W) missense polymorphism regulates the plasma triglyceride levels are not sufficiently well elucidated and need further research. Talmud et al. suggested that the c.56C > G variant may have functional effects on the APOA5 protein [30]. The substitution of hydrophilic serine to hydrophobic tryptophan residue could affect APOA5 signal peptide activity with the endoplasmic reticulum, suggesting that this amino acid change is potentially functional and may reduce the amount of functioning protein [30]. Transgenic and gene knockout mice showed an inverse regulation between APOA5 expression and plasma TG levels, supporting a role for the apolipoprotein A5 in human triglycerides secretion [6].

In the present study, the c.56C > G variant showed significant association with plasma triglyceride levels. We observed elevated triglyceride levels in subjects carrying the GG genotype compared to noncarriers. Similarly, TG levels were also significantly higher in Turkish subjects carrying the GG genotype [43]. The rare allele of the c.56C > G polymorphism was significantly associated with increased TG levels in previous studies on subjects from Spain [44], United Kingdom [30], and Brazil [3].

In contrast, we did not find any relationship between HDL-C levels in subjects carrying the G allele compared to noncarriers, which is consistent with findings reported by other studies [29, 33, 36]. A recent study showed that elevated levels of fasting TG are significantly associated with increased risk of myocardial infarction and gradually decreased levels of HDL-C [45]. Despite this negative correlation that have been shown between HDL-C and plasma TG in several studies, there was no evidence of any association between decreased HDL-C levels and the APOA5 G allele [35].

The association between the c.56C > G polymorphism and susceptibility to CAD remains controversial. To elucidate this discrepancy, we performed a meta-analysis considering the data from all studies exploring the association between this APAO5 variant and increased risk of CAD in various ethnicities.

Several studies were interested in gathering data on APOA5 polymorphisms implication in CAD susceptibility [18, 46–50]. Our meta-analysis showed a strong association between the c.56C > G polymorphism and the risk of CAD under the recessive and homozygote codominant genetic models. Similarly, a previous meta-analysis based on 5 association studies reported a strong association of the c.56C > G polymorphism and genetic predisposition to CAD under the recessive genetic model [46]. In contrast, Zhou and colleagues performed a meta-analysis including 7 studies, and they did not find any significant implication of the c.56C > G variant in CAD susceptibility [19].

Our work may have some limitations. Firstly, CAD is a multifactorial and polygenic disorder involving complex genes and environmental interactions that were not evaluated. Secondly, articles in other languages than English were not included in this meta-analysis. That may result in a bias, although funnel plots and Egger's test showed no significant bias. Lastly, we performed a comprehensive literature research to collect all eligible studies; however, the number of included studies was not large. Thus, further genetic association studies are needed to evaluate the relationship between this APOA5 variant and CAD risk in different populations.

## 5. Conclusion

In summary, the current case-control study showed that the 56C > G polymorphism is a CAD risk factor in the Moroccan population. In addition, our results clarify that the overall conclusion of the literature to data indicates a significant association between 56C > G polymorphism and CAD in different populations, supporting our case-control study results. An improved case-control investigation with larger sample sizes and a balanced gender structure could provide sufficient statistical power to obtain a clear conclusion on the contribution of the 56C > G APOA5 polymorphism in CAD susceptibility.

## Figures and Tables

**Table 1:** Comparison of clinical and biochemical characteristics between controls and patients with CAD.

	Controls (*n* = 134)	CAD (*n* = 122)	*P* value
Age (years)	50.78 ± 12.08	60.70 ± 12.86	<0.001
Sbp (mmgHg)	122 [118–126]	140 [123–160]	<0.001
Dbp (mmgHg)	77.49 ± 7.97	80.83 ± 12.99	0.015
BMI (kg/m^2^)	26.49 ± 4.11	25.68 ± 4.73	0.148
TG (mg/dl)	97 [71.25–122.5]	156 [105.5–179.2]	<0.001
LDL-C (mg/dl)	115 ± 28	141 ± 46	<0.001
HDL-C (mg/dl)	51 ± 13	50 ± 18	0.660
Gly (mg/dl)	89 [85–99]	119 [94–153]	<0.001
TC (mg/dl)	188 ± 28	205 ± 53	0.002
Diabetes (%)	0 (0%)	38 (31%)	<0.001
Smoking (%)	0 (0%)	21 (31%)	<0.001

Sbp: systolic blood pressure, Dbp diastolic blood pressure, BMI: body mass index, TG: Triglycerides, LDL: low-density lipoprotein cholesterol, HDL: high-density lipoprotein cholesterol, Gly: glycemia, TC: serum total cholesterol. Data are expressed as mean ± standard deviation or median (interquartile range) for quantitative parameters and as frequencies for qualitative parameters.

**Table 2:** Genotypic distribution of the 56C > G variant in patients with coronary artery disease and control subjects.

Genetic model	Controls *n* (%)	Cases *n* (%)	Codominant		Dominant		Recessive		Log-additive	
			OR (95% CI)	*P* value	OR (95% CI)	*P* value	OR (95% CI)	*P* value	OR (95% CI)	*P* value
C/C	117 (87.3)	93 (76.2)	1.00	0.001	1.90 (0.87–4.15)	0.107	13.14 (2.56–67.47)	<0.001	2.10 (1.19–3.69)	0.008
C/G	15 (11.2)	13 (10.7)	0.80 [0.30–2.11]							
G/G	2 (1.5)	16 (13.1)	12.85 [2.49–66.22]							
MAF	0.071	0.184								
HWE *P*value	0.125	<0.001								

MAF: minor allele frequency, HWE: *P* value for Hardy–Weinberg equilibrium.

**Table 3:** Association between APOA5 variant genotypes and clinical and biochemical parameters.

Parameter	Genotypes			*P* value	*P* value	*P* value	*P* value
	CC	CG	GG	(CC vs. CG)	(CC vs. GG)	(CC vs. CG + GG)	(CC + CG vs. GG)
	(*n* = 210)	(*n* = 28)	(*n* = 18)				
Dbp (mmgHg)	78.57 ± 9.78	79.18 ± 16.79	84.94 ± 8.73	0.779	0.008	0.102	0.016
Sbp (mmgHg)	123 [119–133]	127 [120–146]	147 [135–175]	0.285	<0.001	0.005	<0.001
BMI (kg/m^2^)	26.18 ± 4.22	25.66 ± 4.99	25.93 ± 5.84	0.550	0.821	0.568	0.866
LDL (mg/dl)	126 ± 39	121 ± 35	150 ± 50	0.531	0.064	0.327	0.057
HDL (mg/dl)	51 ± 16	50 ± 13	45 ± 16	0.667	0.131	0.218	0.132
TC (mg/dl)	194 ± 40	192 ± 37	230 ± 64	0.816	0.033	0.125	0.032
TG (mg/dl)	113 [75–151]	105 [76–133]	212 [158–273]	0.477	<0.001	0.021	<0.001
Gly (mg/dl)	98 [88–119]	92 [90–95]	115 [99–181]	0.281	0.018	0.557	0.012

Sbp: systolic blood pressure, Dbp: diastolic blood pressure, BMI: body mass index, TG: triglycerides, LDL: low-density lipoprotein cholesterol, HDL: high-density lipoprotein cholesterol, Gly: glycemia, TC: serum total cholesterol.

**Table 4:** Characteristics of studies included in the meta-analysis.

Study	Population	Subjects		Sex (M/F)		Average age (years)		Genotypes/alleles c.56C > G									
		Cases	Controls	Cases	Controls	Cases	Controls	Cases					Controls				
								CC	CG	GG	C	G	CC	CG	GG	C	G
Hubacek et al. [32]	Caucasians	435	2559	435/0	1191/1368	51.1 ± 7.6	49	369	56	10	794	76	2198	352	9	4748	370
Liu et al. [33]	China	483	502	285/198	276/226	54.2 ± 6.3	54.4 ± 5.8	439	43	1	921	45	502	0	0	1004	0
Ruiz-Narváez [34]	Costa Rica	1703	1703	1260/443	1260/443	58 ± 11	58 ± 11	NA	NA	NA	3079	327	NA	NA	NA	3059	347
Dallongeville et al. [35]	France	429	458	442/0	475/0	35–64	35–64	368	56	5	792	66	414	44	0	872	44
Martinelli et al. [28]	Italy	669	244	544/125	168/76	60.7 ± 9.3	58.7 ± 12.7	605	59	5	1269	69	219	25	0	463	25
Soufi et al. [36]	German	334	167	276/58	109/58	60 ± 10	54 ± 12	286	43	5	615	53	154	13	0	321	13
Prochaska et al. [29]	Brazil	180	170	62.4/37.6	55.9/44.1	60.1 ± 12.5	58.3 ± 11.3	157	23	0	337	23	145	25	0	315	25
Iqbal et al. [26]	Pakistan	192	192	145/47	130/62	55.6 ± 11.1	53.4 ± 12.4	NA	NA	NA	127	257	NA	NA	NA	100	284
Srivastava et al. [37]	India	304	304	259/45	240/64	56.66 ± 12.16	54.35 ± 9.65	174	106	24	454	154	213	79	12	505	103
Kashyap et al. [16]	India	512	272	448/64	162/110	58.1 ± 10.2	50.3 ± 11.1	505	6	0	1016	6	266	6	0	538	6
Wang et al. [38]	USA	342	537	222/120	260/277	39.8 ± 5.1	53.4 ± 12.1	1	25	316	27	657	0	58	479	58	1016
This study	Morocco	122	134	73/49	42/92	51 ± 12.08	61 ± 12.86	93	13	16	199	45	117	15	2	249	19

NA: missing data in the publication.

**Table 5:** Association between c.56C > G variant and risk of CAD in the meta-analysis.

Model	Number of included studies^*∗*^	Test of association			Test of heterogeneity			Publication bias
		OR	95% CI	*P* value	Model	*P* value	*I* ^2^ (%)	*P* value (Egger's test)
Recessive model (CC + CG vs. GG)	10	3.39	[1.77–6.50]	<0.001	Random	0.05	50	0.086
Dominant model (CC vs. CG + GG)	10	1.35	[0.97–1.87]	0.07	Random	0.001	67	0.700
Co-dominant model (CC vs. CG)	10	1.17	[0.84–1.63]	0.35	Random	0.002	65	0.798
Co-dominant model (CC vs. GG)	10	3.96	[2.44–6.45]	<0.001	Fixed	0.31	15	0.914
Allele contrast (C vs. G)	12	1.29	[1–1.68]	0.05	Random	<0.001	80	0.117

^*∗*^Number of included studies includes our case control study data.

## Data Availability

All data used in this study are included within the article, and a supplementary information file of meta-analysis plots is attached.

## Supplementary Materials

Supplementary Materials The funnel plots of the meta-analysis showing the association of the C56G polymorphism with CAD under all studied inheritance models(recessive, dominant, homozygote co-dominant, heterozygote co-dominant and allelic models).
