# Supplementary material for: Association of c.56C > G (rs3135506) Apolipoprotein A5 Gene Polymorphism with Coronary Artery Disease in Moroccan Subjects: A Case-Control Study and an Updated Meta-Analysis
Source: Cardiol Res Pract. 2020 Aug 4;2020:5981971. doi: 10.1155/2020/5981971 (PMC7424381; doi:10.1155/2020/5981971)
Supplement: Supplementary Materials — The funnel plots of the meta-analysis showing the association of the C56G polymorphism with CAD under all studied inheritance models(recessive, dominant, homozygote co-dominant, heterozygote co-dominant and allelic models). [file 5981971.f1.docx]

**
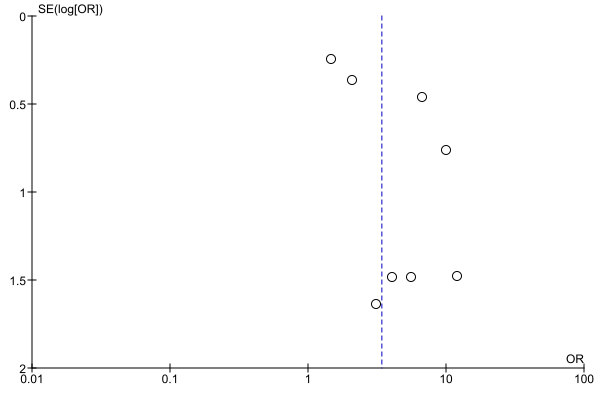
**

**Figure S1 : Funnel plot for the association between c.C56>G gene polymorphism and CAD under recessive model (P=0.086).**

**
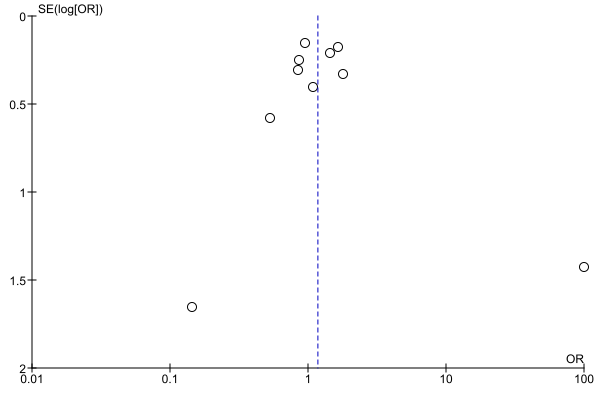
**

**Figure S2 : Funnel plot for the association between c.C56>G gene polymorphism and CAD under heterozygote co-dominant model (P=0.798).**

**
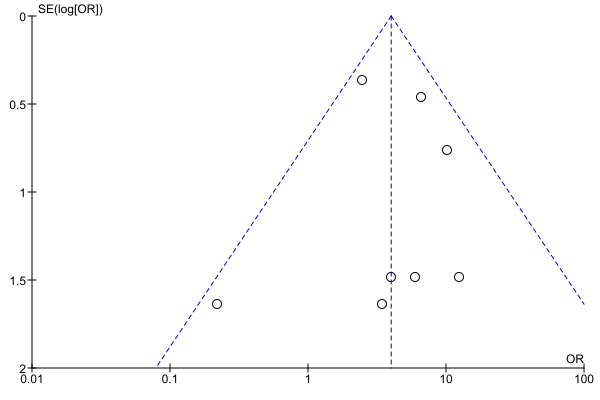
**

**Figure S3 : Funnel plot for the association between c.C56>G gene polymorphism and CAD under homozygote co-dominant model (P=0.914).**

**
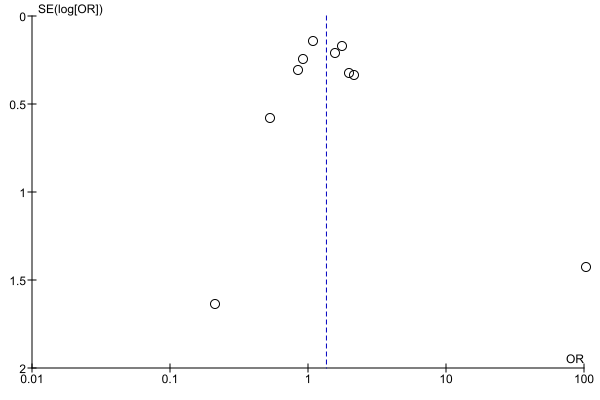
**

**Figure S4 : Funnel plot for the association between c.C56>G gene polymorphism and CAD under dominant model (P=0.700)**

**
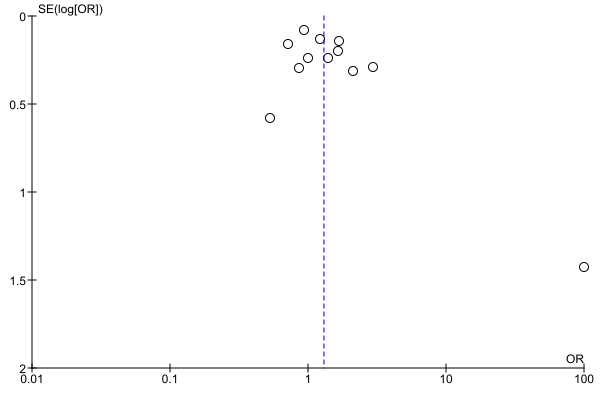
Figure S5 : Funnel plot for the association between c.C56>G gene polymorphism and CAD under allelic model (P=0.117).**
